# Plasma GFAP as a prognostic biomarker of motor subtype in early Parkinson’s disease

**DOI:** 10.1038/s41531-024-00664-8

**Published:** 2024-03-01

**Authors:** Ningning Che, Ruwei Ou, Chunyu Li, Lingyu Zhang, Qianqian Wei, Shichan Wang, Qirui Jiang, Tianmi Yang, Yi Xiao, Junyu Lin, Bi Zhao, Xueping Chen, Huifang Shang

**Affiliations:** Department of Neurology, Laboratory of Neurodegenerative Disorders, National Clinical Research Center for Geriatrics, West China Hospital, Sichuan University, Chengdu, Sichuan China

**Keywords:** Parkinson's disease, Predictive markers

## Abstract

Parkinson’s disease (PD) is a heterogeneous movement disorder with different motor subtypes including tremor dominant (TD), indeterminate and postural instability, and gait disturbance (PIGD) motor subtypes. Plasma glial fibrillary acidic protein (GFAP) was elevated in PD patients and may be regarded as a biomarker for motor and cognitive progression. Here we explore if there was an association between plasma GFAP and different motor subtypes and whether baseline plasma GFAP level can predict motor subtype conversion. Patients with PD classified as TD, PIGD or indeterminate subtypes underwent neurological evaluation at baseline and 2 years follow-up. Plasma GFAP in PD patients and controls were measured using an ultrasensitive single molecule array. The study enrolled 184 PD patients and 95 control subjects. Plasma GFAP levels were significantly higher in the PIGD group compared to the TD group at 2-year follow-up. Finally, 45% of TD patients at baseline had a subtype shift and 85% of PIGD patients at baseline remained as PIGD subtypes at 2 years follow-up. Baseline plasma GFAP levels were significantly higher in TD patients converted to PIGD than non-converters in the baseline TD group. Higher baseline plasma GFAP levels were significantly associated with the TD motor subtype conversion (*OR* = 1.283, *P* = 0.033) and lower baseline plasma GFAP levels in PIGD patients were likely to shift to TD and indeterminate subtype (*OR* = 0.551, *P* = 0.021) after adjusting for confounders. Plasma GFAP may serve as a clinical utility biomarker in differentiating motor subtypes and predicting baseline motor subtypes conversion in PD patients.

## Introduction

Astrocytes constitute a crucial component of the central nervous system. These cells play axon homeostasis and synaptic function^1^. Glial fibrillary acidic protein (GFAP) is considered as a marker of astroglia activation^2^. When the central nervous system is damaged, astrocytes release GFAP into the peripheral blood^1^. Research has demonstrated that astroglia activation contributes to the development of Parkinson’s disease (PD)^3–5^. Several studies indicate that plasma GFAP levels were higher in PD patients compared to healthy controls (HCs)^6^ and were associated with cognitive impairment in PD patients^7,8^. Our previous study has suggested that plasma GFAP could serve as a biomarker for monitoring and predicting motor symptoms progression and cognitive function deterioration in PD^9^. Higher baseline plasma GFAP predicted a more rapid progression to postural instability evaluated with H&Y stage^9^. However, the association between plasma GFAP and motor subtypes, and whether plasma GFAP could predict the conversion of motor subtypes in PD were not explored in previous study^9^.

PD is a clinically heterogeneous disease with patients exhibiting a diverse array of motor and non-motor symptoms^10,11^. Based on clinical symptoms and disease progression, various clinical classification subtypes exist. Among these, tremor dominant (TD), postural instability and gait disturbance (PIGD), and indeterminate subtypes are the most common subtypes in clinical settings and research^12,13^. PIGD subtypes usually had more severe cognitive impairment, faster disease progression, and poor quality of life^14–16^. TD subtypes respond effectively to dopaminergic drugs and exhibit slower disease progression^17^. Motor subtypes are unstable and can transform into one another as the disease progresses^18,19^. Research indicates that approximately 50% of PD patients experience motor subtype conversion within the first two years^18^, with most TD subtypes converting to PIGD and indeterminate subtypes, while PIGD subtypes show limited conversion to other subtypes^19^.

AD co-pathology is frequently found in postmortem examinations of individuals with PD, particularly those who develop dementia and dementia with Lewy bodies (DLB)^20^. A study indicated that the PIGD motor subtype had higher cortical Lewy bodies and a greater burden of cortical amyloid-β plaque compared to the TD motor subtype in PD^21^. PIGD motor subtype was associated with AD co-pathology in DLB^22^. Autopsy indicated that plasma GFAP may serve as a sensitive biomarker for concomitant AD pathology in Lewy body spectrum disorders^23^.

At present, the diagnosis of motor subtypes is based mainly on clinical evaluation, and reliable biomarkers to differentiate motor subtypes are lacking, especially the biomarker of predicting motor subtype conversion. Whether plasma GFAP can be used as a potential biomarker to distinguish motor subtypes and predict motor subtype conversion remains unknown. Thus, we measured plasma GFAP in a longitudinal Chinese PD patients cohort including TD, PIGD, and indeterminate motor subtypes and explored the difference between different subtypes at baseline and over time. We also investigated the relationship between baseline levels of GFAP and motor subtype conversion. Furthermore, we compared levels of plasma GFAP with other blood markers, that is, neurofilament light chain (NfL), p-tau181, Aβ42, and Aβ40.

## Results

### Clinical and demographic profiles

In the study, 184 PD patients and 95 HCs were included. At baseline 184 patients were enrolled, and the enrolled 184 patients had clinical and plasma data at the 1-year follow-up. The enrolled 175 patients (one patient died and eight patients were lost to follow-up) had clinical data and the enrolled 125 patients had plasma data at the 2-year follow-up. The age of the PD patients was 57.78 ± 11.17 years old with an age of onset of 55.81 ± 11.55 years, a disease duration of 1.89 (1.75) years, and a H-Y stage of 2 (0). In the control group (n = 95), 51.6% were male, with a mean age of 55.07 ± 7.31 years. Among the total patients, 62 (33.69%) were classified into the TD subtype, 84 (45.65%) with the PIGD subtype, and 38 (20.65%) had an indeterminate subtype. As shown in Table 1, there were no significant differences in the age, sex, age of onset, disease duration, LEDD, UPDRS-I, UPDRS-II, UPDRS-III, MoCA scores, and PDQ39 scores among the different motor subtypes at baseline. H-Y stage was significantly higher in the PIGD group compared to the TD group (2 (0) vs. 2 (0), *Z* = 2.723, *P* = 0.006). MoCA scores were lower in the PIGD group than in the TD group, but no statistical difference at the 2-year follow-up (25.00 (5) vs. 26.00 (4), *t* = 1.596, *P* = 0.113). UPDRS-III scores were significantly higher in the PIGD group compared to the TD group at 2-year follow-up (32.83 ± 10.15 vs. 28.34 ± 8.54, *t* = 2.562, *P* = 0.011) (Supplementary Table 1).

**Table 1:** Demographic and clinical characteristics of participants at baseline

	HC	PD	TD	Indeterminate	PIGD
Subjects	95	184	62	38	84
Age, years	55.07 ± 7.31	57.78 ± 11.17	57.38 ± 10.39	58.26 ± 10.67	57.87 ± 12.04
Sex M/F	49/46	99/85	35/27	17/21	47/37
Age at onset, years	NA	55.81 ± 11.55	55.52 ± 10.89	56.46 ± 10.76	55.73 ± 12.46
Disease duration, years	NA	1.89 (1.75)	1.72 (1.82)	1.65 (1.52)	1.99 (1.93)
H-Y staging	NA	2 (0)	2 (0)	2 (0)	2 (0)^b**^
LEDD, mg	NA	200.00 (400.00)	188.74 ± 243.61	275.00 (412.50)	250.00 (437.50)
UPDRS-I	NA	0.99 ± 1.59	0 (1)	1.03 ± 1.70	1.12 ± 1.59
UPDRS-II	NA	5.91 ± 4.18	6.08 ± 4.39	4.68 ± 3.63	6.35 ± 4.18
UPDRS-III	NA	22.96 ± 8.85	22.34 ± 8.71	20.92 ± 8.04	24.35 ± 8.95
MoCA	NA	26.00 (4)	27.00 (4)	27.00 (5)	26.00 (3)
PDQ-39	NA	20.74 ± 16.69	19.45 ± 17.08	20.37 ± 14.48	21.86 ± 17.42
Plasma GFAP	57.89 ± 23.54^a*^	69.79 ± 36.18	68.14 (45.92)	66.68 ± 32.75	69.83 ± 38.31
Plasma NfL	8.13 ± 4.29^a***^	10.43 ± 5.76	10.34 ± 5.82	8.62 (7.25)	10.40 ± 5.32
Aβ40	88.14 ± 14.45^a***^	95.01 ± 16.01	95.96 ± 16.68	94.15 ± 15.63	91.72 (16.17)
Aβ42	6.83 ± 1.66^a**^	7.53 ± 1.74	7.47 ± 1.54	7.34 ± 1.85	7.45 (1.76)
Aβ42/Aβ40	0.080 (0.013)	0.079 ± 0.015	0.078 ± 0.012	0.077 ± 0.012	0.082 (0.012)
p-tau181	1.50 ± 0.77	1.64 ± 0.87	1.39 (0.69)	1.72 ± 0.79	1.64 ± 0.88

Data are expressed as mean ± SD or median (interquartile range). Analysis of variance (ANOVA) with Bonferroni as post hoc test and Kruskal–Wallis test were used for multi-group comparison. T-test and Wilcoxon test were used for two-group comparison. Categorical variables were compared with Chi-square tests or Fisher’s exact tests.

*NA* not available, *TD* tremor dominant, *PIGD* postural instability and gait disturbance, *UPDRS* unified Parkinson’s disease rating scale, *LEDD* levodopa equivalent daily dose, *MoCA* Montreal cognitive assessment, *PDQ-39* the 39-item Parkinson’s disease questionnaire, *GFAP* Glial fibrillary acidic protein, *NfL* neurofilament light chain.

*P < 0.05; **P < 0.01; ***P < 0.001.

^a^Differences between Controls and PD.

^b^Differences between TD and PIGD.

### Plasma GFAP between patients with different motor subtypes of PD

**Fig. 1:**
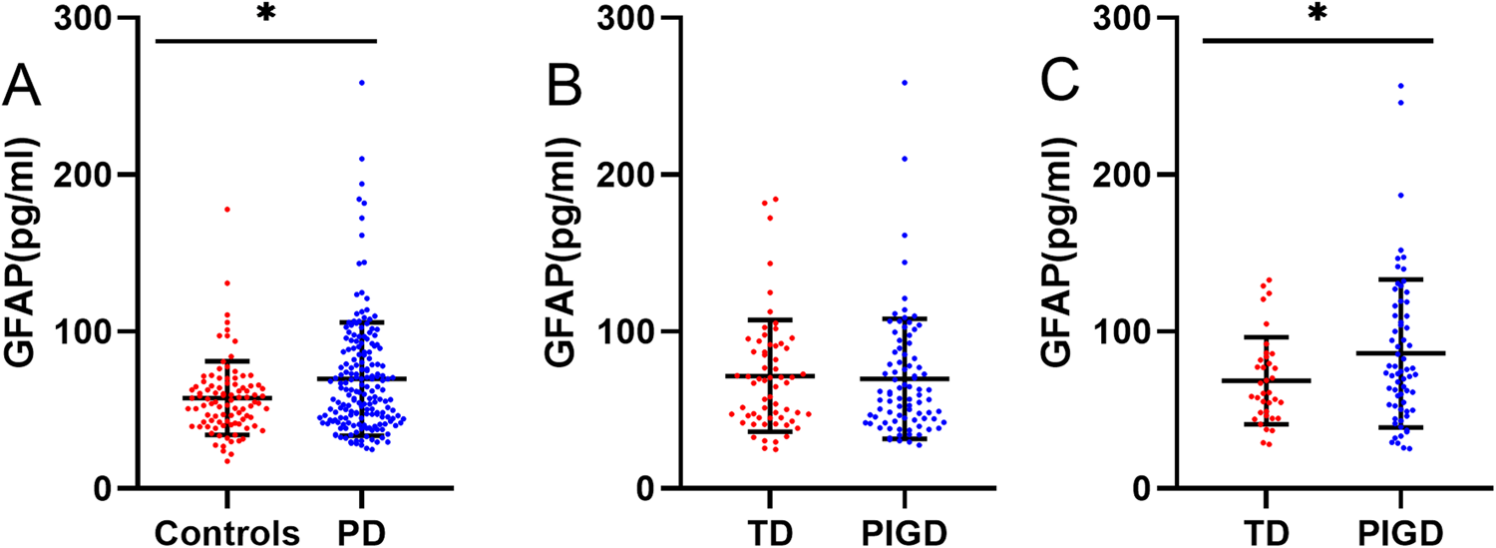
Plasma GFAP in PD patients with different motor subtypes. Plasma GFAP in PD patients and HCs were compared at baseline (**A**). Plasma GFAP in TD and PIGD groups were compared at baseline (**B**) and two-year follow-up (**C**). T-test and Wilcoxon test were used for group comparison. Errors bars represent mean ± standard deviation (*P < 0.05). GFAP Plasma Glial fibrillary acidic protein, PD Parkinson’s disease, TD tremor dominant, PIGD postural instability and gait disturbance.

We found baseline plasma GFAP levels were significantly elevated in PD than that of HCs (69.80 ± 36.18 pg/mL vs. 57.89 ± 23.54 pg/mL, *P* = 0.016) (Fig. 1A). However, baseline plasma GFAP levels were not significantly different between TD and PIGD motor subgroups (68.14 (45.92) pg/mL vs. 69.83 ± 38.31 pg/mL, *Z* = 0.471, *P* = 0.638) (Fig. 1B). Interestingly, we found that plasma GFAP levels of the PIGD subgroup were significantly higher than that of the TD subgroup at the two-year follow-up time point (86.01 ± 47.10 pg/mL vs. 68.57 ± 27.69 pg/mL, *t* = −2.035, *P* = 0.045) (Fig. 1C). Similarly, baseline plasma NFL levels were no difference between TD and PIGD subtypes, but plasma NFL levels of the PIGD subgroup were significantly higher than that of the TD subgroup at a two-year follow-up time point (14.26 ± 10.00 pg/mL vs 10.37 ± 3.93 pg/mL, *t* = −2.223, *P* = 0.028). There were no significant differences in the plasma p-tau181, Aβ42, and Aβ40 between TD and PIGD subtypes at baseline and two-year follow-up time points (Supplementary Table 2).

### Correlations between plasma GFAP and clinical characteristics at baseline

In all PD patients, plasma GFAP was significantly associated with age (*r* = 0.628, *P* < 0.001, duration (*r* = −0.189, *P* = 0.010), and MoCA (*r* = −0.151, *P* = 0.040). There were no associations between plasma GFAP and UPDRS-III (*r* = 0.095, *P* = 0.199), H-Y (*r* = 0.083, *P* = 0.265), and LEDD (*r* = 0.002, *P* = 0.975). In the PIGD group, plasma GFAP was correlated with age (*r* = 0.630, *P* < 0.001) and duration (*r* = −0.249, *P* = 0.023). In the TD group, only age was associated with plasma GFAP (*r* = 0.640, *P* < 0.001). Plasma GFAP levels were all associated positively with plasma NFL, P-tau181, Aβ42, and Aβ40 at baseline and 2-year follow-up (Supplementary Table 3). Plasma GFAP and NfL levels were positively associated with PIGD scores at 2-year follow-up (Supplementary Table 4).

### The conversion of different motor subtypes at two-year follow-up

**Fig. 2:**
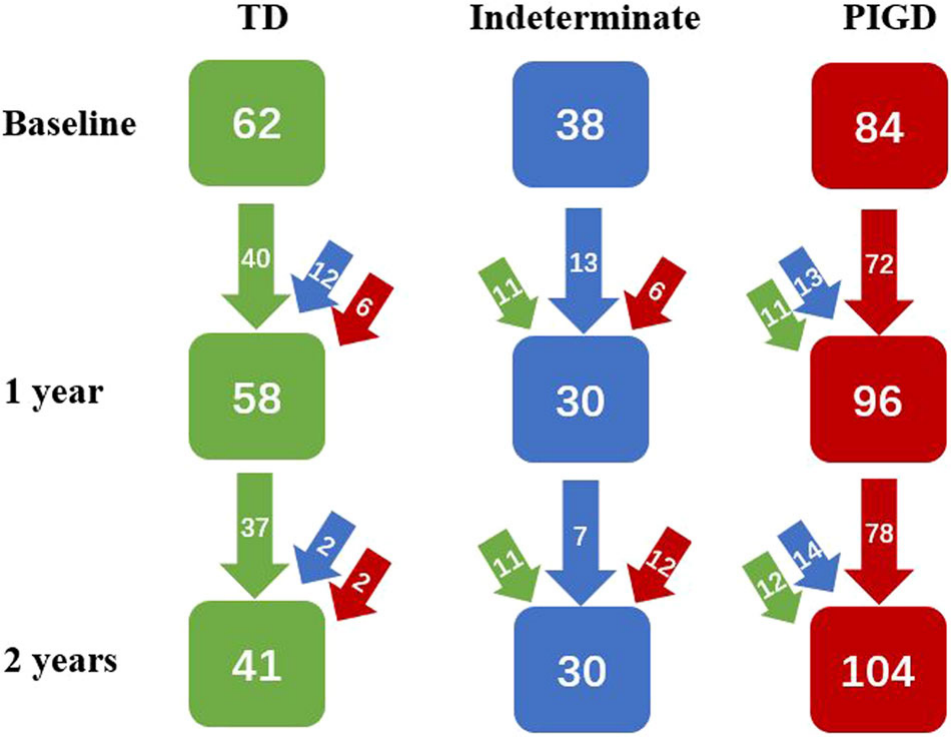
The shift of different motor subtypes over time. Different motor subtypes conversion at 1 year follow-up and 2 years follow-up. TD, indeterminate, and PIGD subtypes were represented by green, blue, and red, respectively. The number of patient subtype conversions was shown in the row. TD tremor dominant, PIGD postural instability and gait disturbance.

Among 184 PD patients, 184 (100%) and 175 (95.1%) patients were assessed at 1-year follow-up and 2-year follow-up, respectively. Therefore, 175 patients (41 TD, 30 indeterminate, and 104 PIGD) with available follow-up data of 2 years were included in subsequent analysis to evaluate the prediction capacity of plasma GFAP levels. The shift of different motor subtypes is shown in Fig. 2. Longitudinally, 30% of baseline TD patients converted to PIGD subtypes, and 15% of them changed to indeterminate subtypes at 2 years follow-up. Finally, 85% of PIGD patients were still categorized as PIGD subtypes at 2 years follow-up, only 4% baseline PIGD patients converted to TD, with 11% shifted to indeterminate subtype. Moreover, 14% of indeterminate patients changed to TD, and 53% changed to PIGD at 2 years follow-up.

### Prediction of motor subtype conversion using baseline plasma GFAP

**Fig. 3:**
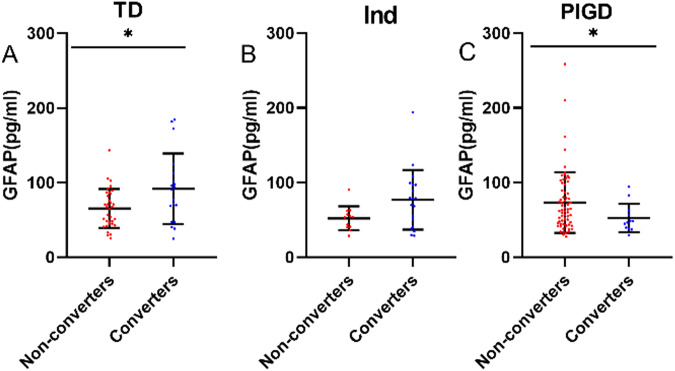
Prediction of motor subtypes conversion using baseline plasma GFAP. Baseline plasma GFAP was compared in non-converters and converters at baseline TD group (**A**), Ind group (**B**), and PIGD group (**C**). T-test and Wilcoxon test were used for group comparison. Errors bars represent mean ± standard deviation (*P < 0.05). GFAP Plasma Glial fibrillary acidic protein, TD tremor dominant, Ind indeterminate, PIGD postural instability and gait disturbance.

Baseline plasma GFAP levels were significantly higher in TD patients converted to PIGD than non-converters in the baseline TD group (91.67 ± 47.39 pg/mL vs. 65.40 ± 25.98 pg/mL, *t* = 2.615, *P* = 0.012) (Fig. 3). ROC analysis indicated that plasma GFAP predicted the motor subtype transformation with an AUC of 0.6656, 95% CI: 0.4985–0.8328, sensitivity = 52.63%, specificity = 85.29%). Similarly, we also found that baseline plasma GFAP levels were significantly elevated in indeterminate patients converted to PIGD compared with non-converters in the baseline indeterminate group (78.57(56.67) pg/mL vs. 51.99 ± 16.14 pg/mL, *Z* = 1.784, *P* = 0.074). Baseline plasma GFAP levels were lower in PIGD patients converted to TD and indeterminate subtypes compared to that of non-converters in the baseline PIGD group (48.07 ± 14.11 pg/mL vs. 74.07 ± 40.49 pg/mL, *t* = 2.191, *P* = 0.032). ROC analysis indicated that plasma GFAP predicted the PIGD subtype transformation with an AUC of 0.7376, 95% CI: 0.6092–0.8659, sensitivity=71.64%, specificity=66.67%). Plasma GFAP levels were significantly higher in TD convert to PIGD group than TD stable group at 2-year follow-up (109.42 ± 59.29 pg/mL vs.70.48 ± 28.72 pg/mL, *t* = 2.811 *P* = 0.008). There were no differences in plasma GFAP levels between non-converters and converters in the Ind group at 2-year follow-up (51.49 ± 12.56 vs 70.44 ± 25.66, *t* = 1.846 *P* = 0.078). Plasma GFAP levels were higher in PIGD stable group than PIGD convert to TD group at 2-year follow-up (71.27 (56.84) vs 45.47 ± 11.97, *Z* = 2.846 *P* = 0.004) (Supplementary Fig. 1). No significant differences were observed at the baseline plasma NFL, p-tau181, Aβ42, and Aβ40 levels among different motor subtype conversions (Supplementary Table 5).

**Fig. 4:**
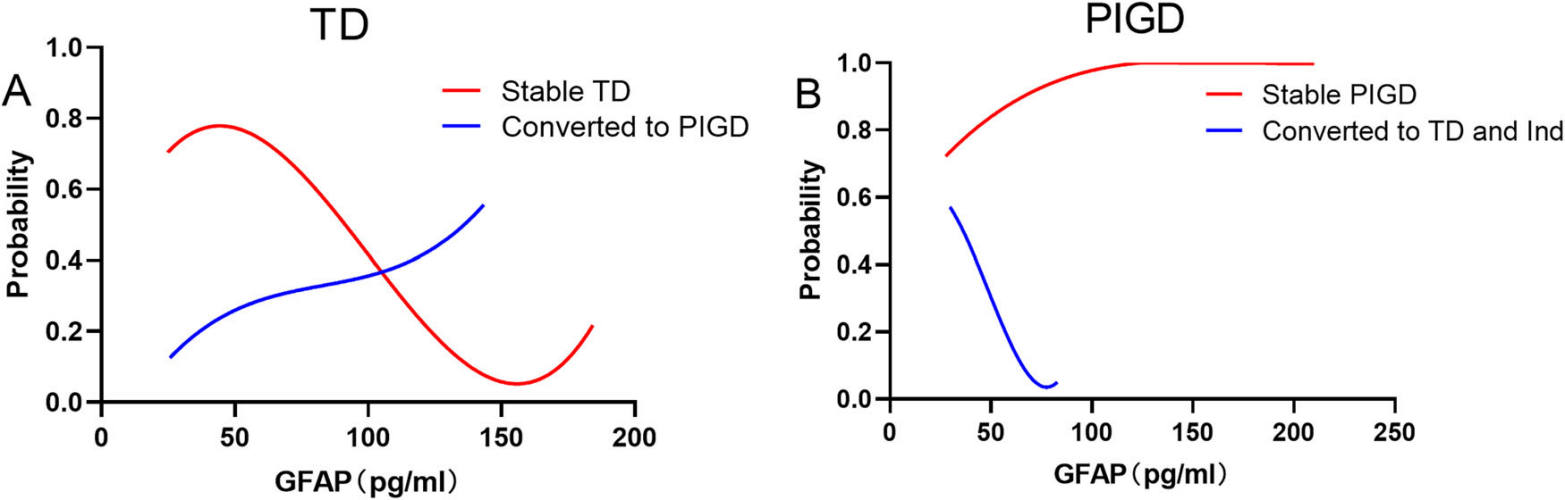
Baseline plasma GFAP in prediction of motor subtypes after correction for confounders. Relationship between baseline plasma GFAP and the probability of TD (**A**) and PIGD (**B**) motor subtypes after correction for confounders. Predicted probabilities for stable subtypes were represented with a red line. Predicted probabilities of motor subtype converters were shown in the blue line. GFAP Plasma Glial fibrillary acidic protein, TD tremor dominant, PIGD postural instability and gait disturbance.

The logistic regression analyses between baseline plasma GFAP levels and motor subtype conversion are summarized in Table 2. Univariate logistic regression analysis with TD motor subtype conversion as the dependent variable showed a significant association with baseline plasma GFAP (*OR* = 1.251, *P* = 0.027). PIGD motor subtype conversion was correlated significantly with baseline plasma GFAP (*OR* = 0.614, *P* = 0.017). To better explore the independent association between baseline plasma GFAP and motor subtype conversion, a multivariate logistic regression model was applied with non-converters in baseline TD and PIGD groups as the reference. Overall, a higher baseline GFAP was significantly associated with the TD motor subtype conversion (*OR* = 1.283, *P* = 0.033) after adjusting for age and UPDRS-III. With every 10 units increasing baseline plasma GFAP levels, TD patients were 28.3% more likely to convert to the PIGD subtype (Fig. 4A). Similarly, a lower baseline GFAP was related to the PIGD motor subtype shift (*OR* = 0.551, *P* = 0.021) after adjusting for age, H-Y, and UPDRS-III. Every 10 units decreasing baseline plasma GFAP levels, PIGD patients were 45.9% more likely to convert to the TD and indeterminate subtypes. (Fig. 4B).

**Table 2:** Multivariate logistic regression analysis for the independent association between baseline plasma GFAP and motor subtype conversions

Motor subtypes	Univariate^a^		Multivariate^b^	
	OR (95% CI)	*P* value	OR (95% CI)	*P* value
GFAP				
TD group	1.251 (1.025–1.525)	0.027	1.283 (1.020–1.614)	0.033
PIGD group	0.614 (0.412–0.917)	0.017	0.551 (0.332–0.915)	0.021

*CI* confidence interval, *OR* odds ratio, *GFAP* Glial fibrillary acidic protein, *PIGD* postural instability and gait disturbance, *TD* tremor-dominant, *PIGD* postural instability and gait disturbance, *UPDRS* Unified Parkinson’s Disease Rating Scale.

^a^Results from univariable logistic regression.

^b^Results from multivariable logistic regression: TD group adjusted for age and UPDRS-III; PIGD group adjusted for age, H-Y, and UPDRS-III.

### Plasma GFAP, cognitive and motor data in PD patients over different time points

Longitudinal data of plasma GFAP, cognitive, and motor scores are summarized in Table 3. In the total PD patients, plasma GFAP did not elevate significantly over time (*P* = 0.560). Similarly, no significant difference in plasma GFAP was observed in the TD group (*P* = 0.901) and indeterminate group (*P* = 0.057) over time. However, plasma GFAP levels were significantly increased over time in the PIGD group (*P* = 0.038).

**Table 3:** Plasma GFAP, cognitive and motor data in PD patients over different timepoints

Group	Values	Time			Trend *P* value
		Baseline	1 year	2 years	
Total PD	GFAP	69.79 ± 36.18	72.28 ± 38.59	74.53 ± 40.38	0.560
	MoCA	26.00 (4)	26.00 (4)	25.00 (5)	0.058
	UPDRS-III	22.96 ± 8.75	26.87 ± 9.07	30.55 ± 9.73	0.000
TD	GFAP	68.14 (45.92)	70.54 ± 30.91	68.57 ± 27.69	0.969
	MoCA	27.00 (4)	26.00 (4)	26.00 (4)	0.812
	UPDRS-III	22.34 ± 8.71	23.38 ± 7.96	28.34 ± 8.54	0.001
Ind	GFAP	66.68 ± 32.75	60.05 (35.76)	49.79 ± 16.99	0.047
	MoCA	27.00 (5)	27.00 (4)	26.00 (5)	0.244
	UPDRS-III	20.92 ± 8.04	25.37 ± 7.61	26.32 ± 7.88	0.011
PIGD	GFAP	69.83 ± 38.30	72.23 ± 37.92	86.01 ± 47.10	0.038
	MoCA	26.00 (3)	25.00 (5)	25.00 (5)	0.181
	UPDRS-III	24.35 ± 8.95	29.45 ± 9.38	32.83 ± 10.15	0.000

Data are expressed as mean ± SD or median (interquartile range). T-test and Wilcoxon test were used for two-group comparison.

*PD* Parkinson’ s disease, *GFAP* Glial fibrillary acidic protein, *TD* tremor dominant, *PIGD* postural instability, and gait disturbance, *UPDRS* unified Parkinson’s disease rating scale, *MoCA* Montreal Cognitive Assessment.

## Discussion

In the study, we showed that plasma GFAP levels were higher in PIGD subtypes compared to TD subtypes over two years. Additionally, we demonstrated that plasma GFAP levels were significantly elevated over time in the PIGD subtypes, but not in TD subtypes. Moreover, our study suggested that higher baseline plasma GFAP levels in the TD group can predict the conversion of TD motor subtypes to PIGD subtypes, and lower baseline plasma GFAP levels in the PIGD group can predict the conversion to TD and indeterminate subtypes within a relatively short follow-up period of two years.

GFAP serves as a biomarker of reactive astrogliosis, exhibiting elevated levels in plasma and cerebrospinal fluid (CSF) in neurodegenerative diseases such as Alzheimer’s disease (AD)^24,25^, dementia with Lewy bodies^26^, frontotemporal lobar degeneration^27^, and PD^28^. Plasma GFAP levels not only increased in AD but also raised in preclinical AD compared to cognitively unimpaired older adults^24^. A recent study suggests that the magnitude change of plasma GFAP levels was higher than that in CSF in AD patients^29^. These results indicate that plasma GFAP could serve as a convenient, sensitive biomarker for detecting reactive astrogliosis. Therefore, dynamic changes in plasma GFAP of PD patients were measured during a two-year follow-up period. Our results showed that plasma GFAP levels of PD patients were higher than those of controls, which was supported by some previous studies^7,8^.

No significant differences in the plasma GFAP levels were observed between PIGD and TD subtypes at baseline, which may be due to both PD subtype patients being in the early disease stage with a median disease duration of 1.89 (1.75) years. The lack of difference in plasma GFPA levels between TD and PIGD at baseline may depend on the fact that each subgroup also includes future converters. Reactive astrogliosis has been observed in the substantia nigra pars compacta of both PD patients and PD animal models, leading to dysfunction in dopaminergic neurons^30,31^. Studies suggested that the akinetic-rigid dominant subtype or PIGD motor subtype experienced more impairment of DA neurons than the TD motor subtype^13,21^. Our study reveals significantly elevated plasma GFAP levels in the PIGD subgroup compared to the TD group at the two-year follow-up, indicating a more intense pathophysiology in PIGD patients.

Previous studies have shown that the PIGD subtype not only exhibited greater cognitive progression, worse hallucinations, and poorer fatigue^14,15,32^ but also increased risk of falls and gait freezing compared to the TD subtype^32,33^. With a poorer prognosis and more rapid disease progression^14^, the PIGD motor subtype could be a reliable predictor of disease progression. Therefore, reliable biomarkers for diagnosing motor subtypes are essential. Our study highlights the importance of plasma GFAP in the PD motor subtype.

In addition to plasma GFAP, other plasma biomarkers were investigated to differentiate motor phenotype in our study. The results suggest that patients with the PIGD subtype exhibited higher plasma NFL levels compared to that of patients with the TD subtype at the two-year follow-up time point, however, no difference at baseline, which was consistent with a previous study^34^. Our results showed that plasma GFAP levels were significantly associated with plasma NFL levels. Plasma NfL is a reliable biomarker for neuronal injury and axonal degeneration and it was associated with the disease severity and progression of PD^35^ and other neurodegenerative disorders^36^. Plasma GFAP showed a significant correlation with plasma NfL in AD^29^ and FTD^37^. Our results indicated that axonal damage and astroglia activation were coexistent in PD, with even greater intensity in the PIGD motor subtype.

In the current study, we found plasma p-tau181, Aβ42, and Aβ40 did not differ between TD and PIGD subgroups at baseline and the two-year follow-up time point. AD co-pathology is frequently found in postmortem examinations of individuals with PD^38^, particularly those who develop dementia and DLB^20^. An autopsy indicated that plasma GFAP may serve as a sensitive biomarker for AD co-pathology in Lewy body spectrum disorders, especially the accumulation of β-amyloid plaques^23^. Our results showed a correlation between plasma GFAP levels and plasma P-tau181, Aβ42, and Aβ40 in PD, indicating an association between plasma GFAP with AD co-pathology in PD.

Research showed that the PIGD motor subtype had higher mean pathological grading of cortical Lewy bodies and more cortical amyloid-β plaque load than the TD motor subtype in PD^21^. A study showed that PIGD scores were related to the CSF t-tau/Aβ1-42 ratio, indicating AD co-pathology may contribute to PIGD motor signs in LBD^22^. Plasma GFAP levels were positively associated with PIGD scores at 2-year follow-up in our study. Plasma GFAP is a marker of astrogliosis, elevation tends to be highest in AD Simone Baiardi^39^ and may serve as a sensitive biomarker for concomitant AD pathology in LBD^23^. Therefore, we speculated that AD co-pathology may contribute to PIGD motor symptoms in PD. Besides, AD co-pathology may also contribute to worse cognition and faster disease progression in the PIGD motor subtype in PD. Reliable biomarkers for diagnosing motor subtypes are essential. Our study highlights the importance of plasma GFAP and AD co-pathology in the PD motor subtype.

In addition to plasma Aβ42 and Aβ40, the Aβ42/Aβ40 ratio was typically used in AD, and the ratio was associated with neocortical amyloid burden^40^. A study showed that APOE-ε4 status was associated with the plasma Aβ42/Aβ40 ratio and cognitive decline in PD^41^. However, there was no difference in the Aβ42/Aβ40 ratio between the TD group and the PIGD group at baseline and 2-year follow-up. Astrocyte reactivity is a crucial initial event linking Aβ and tau pathology in preclinical AD^42^. We speculated that astrocyte reactivity may occur before Aβ and tau pathology in early PD, leading to no difference in the Aβ42/Aβ40 ratio between the TD group and the PIGD group.

One previous study revealed lower plasma Aβ42 levels in the PIGD group than that in the TD group^43^. The inconsistent results may be due to the disease duration being approximately 4 years in the previous study^43^. A study from the Parkinson’s Progression Markers Initiative (PPMI) cohort found that α-synuclein, Aβ1-42 and p-tau181 levels in CSF were lower in patients with PIGD subtypes than in patients with TD subtypes^44^. The BioFIND study on patients with moderate-advanced PD found that CSF α-synuclein levels were lower in patients with the PIGD subtype than in patients with other motor subtypes^45^. Although these CSF proteins could serve as biomarkers to distinguish different motor subtypes, lumbar puncture is an invasive examination and clinical application is limited. Therefore, reliable and non-invasive tests may be crucial for diagnosing and predicting motor subtypes.

Motor subtypes of PD can be unstable and may shift as the disease progresses. One previous study showed that one-third of the TD subtype at baseline remained the TD subtype during six years of follow-up and most of the patients with TD subtype at baseline converted into other motor subtypes including 15% indeterminate and 50% PIGD subtypes^19^. Our results showed that about one-half of PD patients had a motor subtype conversion at 2 years follow-up, which was also consistent with a previous study^46^. Several studies explored the factors influencing the conversion of baseline TD subtypes^18,47,48^. Research from the PPMI cohort revealed that patients who transitioned from the TD subtype to the PIGD subtype during four years of follow-up exhibited higher UPDRS-II scores, more severe autonomic dysfunction, and a higher proportion of levodopa-induced dyskinesia than non-convertors^48^. A study conducted by Garcıa et al. suggested that motor subtype converters had worse global non-motor symptoms measured by the NMSS, poorer cognitive function, and lower serum levels of methylmalonic acid at baseline than those with stable-TD motor phenotypes during disease progression^49^. However, predictive factors for motor subtype converters are mainly focused on clinical features.

Motor subtype converters experienced faster disease progression and worse clinical prognoses^50^. Lack of studies on biomarkers besides clinical factors that predict the conversion of TD to PIGD subtypes. In the present study, it was demonstrated that higher plasma GFAP in patients with TD subtypes at baseline could shift to the PIGD subtypes at a two-year follow-up. Conversely, lower plasma GFAP levels in patients with PIGD subtypes at baseline could convert to the TD and indeterminate subtypes during follow-up. Similar results were not observed for plasma NFL, p-tau 181, Aβ42, and Aβ40. Therefore, our findings suggest that baseline plasma GFAP levels could serve as a valid biomarker for predicting motor subtype conversion and disease progression in PD.

We acknowledge the limitations of our study. The current study was a single-center design and the sample size was still relatively small. Therefore, future larger and multicenter studies including more patients with the indeterminate subtype are encouraged. The follow-up period was only 2 years, which makes it difficult to conclude the long-term progression of different motor subtypes in PD. Additionally, medication effects on PD motor subtype classification at different follow times should be considered.

In conclusion, PD patients with the PIGD subtype have evidence of higher plasma GFAP compared to the TD subtype. Plasma GFAP may represent a potential biomarker for identifying motor subtypes and predicting the conversion of different motor subtypes in PD.

## Methods

### Participants

Patients with PD were from an ongoing perspective longitudinal cohort study supported at the Department of Neurology of West China Hospital of Sichuan University. All subjects agreed to participate in the study and written informed consent was obtained. The study was approved by the Ethics Committee of West China Hospital of Sichuan University.

One hundred and eighty-four early-stage PD patients (disease duration <3 and Hoehn & Yahr [H&Y] stage ≤3) and 95 healthy controls (HCs) were included in the study. The enrolled 150 PD patients come from our previous study^9^. All subjects were recruited between February 2015 and November 2020 and followed for up to 2 years. PD patients were diagnosed clinically according to the United Kingdom Brain Bank diagnostic criteria^51^. Clinically established PD patients were recruited after follow-up evaluation. Atypical parkinsonism such as progressive supranuclear palsy, multiple system atrophy, dementia with Lewy bodies, and secondary parkinsonism (vascular, drug-induced, inflammatory, immune-mediated, infectious, traumatic, etc.) was excluded. HCs were recruited from volunteers or spouses of PD patients who had no history of neurological diseases and other severe disorders.

### Clinical evaluation

The detailed data collection method was described in our previous study^11^. Age, sex and education level were collected in all subjects at baseline. Age of onset and disease duration of PD patients were collected. The motor and non-motor symptoms of PD patients were carefully evaluated by experienced movement disorder specialists at baseline and every 12 months during the follow-up period. The assessment of motor was performed in the “OFF” state and the evaluation of non-motor symptoms including cognitive function was performed in the “ON” state in PD patients. PD symptoms were assessed with part I, II, and III sub-scales of the Movement Disorders Society Unified Parkinson’s Disease Rating Scale (MDS-UPDRS) and H-Y staging was undertaken for all PD patients. Montreal Cognitive Assessment (MoCA, Beijing Version) was used to assess cognitive function. Quality of life was evaluated with the 39-item Parkinson’s disease questionnaire (PDQ-39) with a higher score indicating a poorer quality of life. Levodopa equivalent daily dose (LEDD) was assessed according to the levodopa conversion formula^52^. Briefly, 100 mg levodopa = 133 mg entacapone = 1 mg pramipexole = 5 mg ropinirole = 10 mg selegiline = 1 mg rasagiline = 100 mg amantadine.

### Motor subtypes

The motor subtype classification of PD patients was evaluated according to Stebbins et al.^53^. Briefly, the ratio of the mean MDS-UPDRS tremor scores (11 items) to the mean MDS-UPDRS PIGD scores (5 items) was calculated for each patient. If the ratio was ≤0.90, patients were classified as a PIGD subtype, if the ratio was ≥1.15 as a TD subtype, and if the ratio was between 0.90 and 1.15 as an indeterminate subtype. Motor subtypes were evaluated repeatedly at the 1-year and 2-year follow-up time points. Converters were grouped during follow-up according to TD/PIGD groupings at baseline.

### Measurement of plasma GFAP, NFL, Aβ40, Aβ42, and p-Tau181

Fasting venous blood of 4 ml whole blood (EDTA anticoagulant tube) was collected from all PD patients and healthy controls, centrifuged at 4 °C within 2 h (2000 rpm, 10 min), the upper plasma was collected and stored in the refrigerator at −80 °C to avoid repeated freezing and thawing. Plasma GFAP, NfL, Aβ40, Aβ42, and p-tau181 were measured for all participants at baseline, 1-year, and 2-years follow-up using an ultrasensitive single-molecule array (Simoa^TM^; Quanterix, Billerica, MA, USA) on the automated Simoa^TM^ HD-X platform (GBIO, Hangzhou, China). The Neurology 4-Plex E Assay Kit (Cat No:103670) and p-tau181 Advantage V2 Assay Kit (Cat No:103714) were used (Quanterix). The plasma sample was randomized, blinded, and measured using a batch of reagents from the same lot.

### Statistical analysis

The Shapiro–Wilk test was applied to assess the normality of data. For normal variables, numbers are expressed as mean ± standard deviation (SD). Analysis of variance with Bonferroni as post hoc test and t-tests were used for group comparison. For non-normal or non-homoscedasticity variables, numbers are expressed as median (interquartile range). Multiple comparisons were performed by the non-parametric Kruskal–Wallis test. Categorical variables were compared by Chi-square tests and Fisher’s exact tests.

Spearman correlation analysis was performed to assess the association between baseline plasma GFAP levels and clinical characteristics in different groups.

After adjusting for age, factors that affect motor subtype transformation were investigated by logistic regression analysis. Variables for inclusion were carefully selected, considering the number of events available and the univariate relationship. The receiver operating characteristic (ROC) curve was used to analyze the capability of baseline plasma GFAP levels for predicting the shift of the baseline TD subtype.

All analyses were carried out using SPSS version 22.0 (IBM Corporation, Armonk, NY, USA). Scatter plots and ROC curves were generated using GraphPad Prism version 8.0 (GraphPad Software, Inc, San Diego, CA, USA). *P* < 0.05 was considered statistically significant.

### Supplementary information

Supplementary Information

nr-reporting-summary

## Data Availability

The authors confirm that the data supporting the findings of this study are available within the article.
