# Supplementary material for: Plasma GFAP as a prognostic biomarker of motor subtype in early Parkinson’s disease
Source: NPJ Parkinsons Dis. 2024 Mar 1;10:48. doi: 10.1038/s41531-024-00664-8 (PMC10907600; doi:10.1038/s41531-024-00664-8)
Supplement: Supplementary file 1 — Supplementary Information [file 41531_2024_664_MOESM1_ESM.pdf]

Supplementary Information

Plasma GFAP as a prognostic biomarker of motor subtype in early Parkinson's disease

Ningning Che et al.

Contents

Supplementary Figures

Supplementary Figure 1. Plasma GFAP in non-converters and convertors at 2-year follow-up

Supplementary Tables

Supplementary Table 1. Clinical characteristics in PD patients at 2-year follow-up.

Supplementary Table 2. Plasma biomarkers in PD patients over different time points

Supplementary Table 3. The associations between plasma GFAP and other biomarkers at baseline and 2-year follow-up.

Supplementary Table 4. The associations between plasma biomarkers and the TD scores and PIGD scores

Supplementary Table 5. Baseline plasma biomarkers among different motor subtype conversions.

Supplementary Figures

Supplementary Figure 1. Plasma GFAP in non-converters and convertors at 2-year follow-up

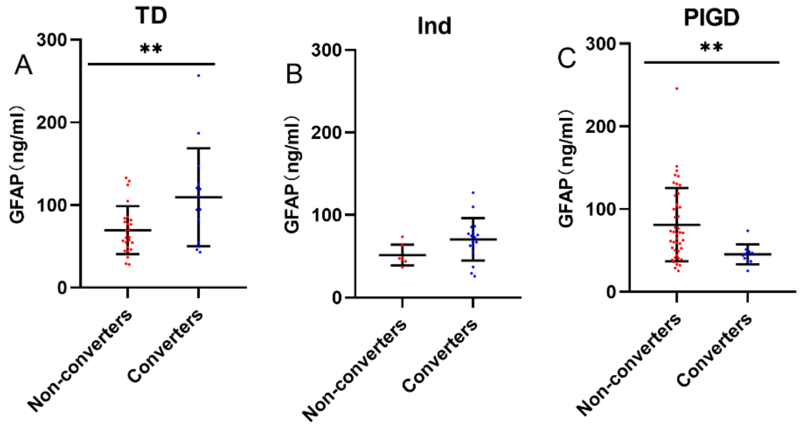

Plasma GFAP was compared in non-converters and converters at TD group (A), Ind group (B), and PIGD group (C) at 2-year follow-up. T-test and Wilcoxon test were used for group comparison. Errors bars represent mean ± standard deviation (*$P < 0.05$, **$P < 0.01$). Abbreviations: GFAP, Plasma Glial fibrillary acidic protein; TD, tremor dominant; Ind, indeterminate; PIGD, postural instability and gait disturbance.

Supplementary Tables

Supplementary Table 1. Clinical characteristics in PD patients at the 2-year follow-up.

	PD	TD	Indeterminate	PIGD	*P*
**Subjects**	175	41	30	104	
**Age**	60.09±11.35	59.58±10.27	57.94±10.70	60.98±11.97	0.408
**Sex M/F**	94/81	25/16	17/13	52/53	0.218
**Disease duration, years**	3.97 (1.79)	4.02±1.23	3.63 (1.33)	4.11 (11.92)	0.415
**H-Y staging**	2.00 (0)	2.00 (0)	2.00 (0)	2.00 (0.5)	<0.001
**LEDD, mg**	450.00 (300.00)	300.00 (287.50)	375.00 (225.00)	450.00 (275.00)	<0.001
**UPDRS-I**	1.54±1.65	1.39±1.21	1.48±1.50	1.62±1.86	0.725
**UPDRS-II**	8.29±4.95	7.00±3.33	6.13±5.01	9.52±5.18	<0.001
**UPDRS-III**	30.55±9.73	28.34±8.54	26.32±7.88	32.83±10.15	<0.001
**MoCA**	25.00 (5)	26.00 (4)	26.00 (5)	25.00 (5)	0.231

Data are expressed as mean ± SD or median (interquartile range). Analysis of variance (ANOVA) with Bonferroni as post hoc test and Kruskal-Wallis test were used for multi-group comparison. Abbreviations: TD, tremor dominant; PIGD, postural instability and gait disturbance; UPDRS, unified Parkinson’s disease rating scale; LEDD, levodopa equivalent daily dose; MoCA, Montreal cognitive assessment.

Supplementary Table 2. Plasma biomarkers in PD patients over different time points

	PD	TD	Indeterminate	PIGD	*P*
**Baseline GFAP**	69.79±36.18	68.14 (45.92)	66.67±32.74	69.83±38.30	0.638
**1 year**	72.28±38.59	70.54±30.91	60.05 (35.76)	72.23±37.92	0.874
**2 years**	74.53±40.38	68.57±27.69	49.78±16.99	86.01±47.10	0.045
**Baseline NFL**	10.43±5.76	10.33±5.82	8.62 (7.25)	10.40±5.31	0.975
**1 year**	11.18±6.19	9.75±3.90	11.65±4.98	9.98 (8.02)	0.334
**2 years**	11.96±8.09	10.37±3.93	7.83±3.17	14.26±10.00	0.028
**Baseline p-tau181**	1.64±0.87	1.39 (0.69)	1.72±0.79	1.63±0.88	0.365
**1 year**	1.83±1.23	1.76±1.41	1.92±1.35	1.83±1.08	0.723
**2 years**	1.92±1.23	1.98±1.64	1.68±0.71	1.95±1.11	0.910
**Baseline Aβ40**	95.01±16.01	95.95±16.68	94.15±15.63	91.72 (16.17)	0.253
**1 year**	94.17±14.42	91.86 (18.13)	97.50±16.14	93.82±15.13	0.337
**2 years**	89.11±18.08	90.86±13.75	80.13±17.08	91.75 (23.26)	0.900
**Baseline Aβ42**	7.53±1.74	7.47±1.54	7.33±1.85	7.45 (1.76)	0.956
**1 year**	7.44±1.61	7.47±1.40	7.69±1.49	7.44 (2.07)	0.556
**2 years**	6.99±1.71	7.10±1.21	6.54 (1.39)	7.16±1.97	0.105
**Baseline Aβ42/40**	0.079±0.015	0.078±0.012	0.077±0.012	0.082 (0.012)	0.453
**1 year**	0.079±0.013	0.084 (0.015)	0.079±0.008	0.080 (0.016)	0.178
**2 years**	0.079±0.016	0.078±0.010	0.080±0.011	0.079 (0.016)	0.738

Data are expressed as mean ± SD or median (interquartile range). Analysis of variance (ANOVA) with Bonferroni as post hoc test and Kruskal-Wallis test were used for multi-group comparison. Abbreviations: PD, Parkinson’ s disease; TD, tremor dominant; PIGD, postural instability and gait disturbance; GFAP, Glial fibrillary acidic protein; NfL: neurofilament light chain.

Supplementary Table 3. The associations between plasma GFAP and other biomarkers at baseline and 2-year follow-up.

	Baseline		1 year		2 years	
	r	*P*	*r*	*P*	*r*	*P*
**NFL**	0.568	<0.001	0.571	<0.001	0.636	<0.001
**P-tau181**	0.303	0.001	0.356	<0.001	0.490	<0.001
**Aβ42**	0.320	<0.001	0.475	<0.001	0.375	<0.001
**Aβ40**	0.425	<0.001	0.561	<0.001	0.529	<0.001

Spearman correlation analysis was performed to assess the associations. Abbreviations: GFAP, Glial fibrillary acidic protein; NfL: neurofilament light chain.

Supplementary Table 4. The associations between plasma biomarkers and the TD scores and PIGD scores

	GFAP		NFL		P-tau181		Aβ42		Aβ40	
	r	***P***	***r***	***P***	***r***	***P***	***r***	***P***	***r***	***P***
**Baseline**										
**TD scores**	0.036	0.630	0.036	0.732	0.023	0.829	-0.070	0.505	-0.097	0.355
**PIGD scores**	0.045	0.546	0.125	0.234	0.065	0.536	0.007	0.950	0.104	0.319
**1 year**										
**TD scores**	0.007	0.927	-0.036	0.630	-0.005	0.946	0.098	0.185	-0.026	0.726
**PIGD scores**	0.066	0.376	0.202	0.006*	0.092	0.213	0.044	0.953	0.067	0.369
**2 years**										
**TD scores**	0.134	0.138	-0.096	0.286	-0.026	0.776	-0.045	0.614	0.054	0.549
**PIGD scores**	0.218	0.015*	0.279	0.002*	0.040	0.658	0.043	0.636	0.173	0.054

Spearman correlation analysis was performed to assess the associations. Abbreviations: TD, tremor dominant; PIGD, postural instability and gait disturbance; GFAP, Glial fibrillary acidic protein; NfL: neurofilament light chain.

Supplementary Table 5. Baseline plasma biomarkers among different motor subtype conversions.

	TD			Indeterminate			PIGD		
	**Non-converters n=33**	**Converters PIGD n=18**	***P***	**Non-converters n=12**	**Converters PIGD n=19**	***P***	**Non-converters n=67**	**Converters TD n=12**	***P***
**GFAP**	65.40±25.98	91.67±47.39	0.012*	51.99±16.14	78.57(56.67)	0.074	73.28±40.61	52.48±19.19	0.032*
**NFL**	10.04±5.52	11.45±7.12	0.425	8.37±3.24	13.49 (8.58)	0.048*	10.58±5.49	8.54 (6.14)	0.633
**p-tau181**	1.42 (0.73)	1.53±0.69	0.941	1.47±0.77	1.89±0.86	0.162	1.68±0.94	1.44±0.41	0.393
**Aβ42**	7.47±1.52	7.56±1.72	0.852	7.03±1.12	7.56±2.36	0.407	7.66±1.99	7.72±1.06	0.913
**Aβ40**	95.47±17.98	99.44±16.06	0.427	93.32±8.24	96.29±17.80	0.594	95.12±17.14	96.57 (11.62)	0.436
**Aβ42/Aβ40**	0.079±0.012	0.074 (9.913)	0.354	0.075±0.012	0.082 (0.026)	0.372	0.081±0.019	0.080±0.009	0.901

Abbreviations: TD, tremor dominant; PIGD, postural instability and gait disturbance; GFAP, Glial fibrillary acidic protein; NfL: neurofilament light chain.
